# IL-17, IL-27, and IL-33: A Novel Axis Linked to Immunological Dysfunction During Sepsis

**DOI:** 10.3389/fimmu.2019.01982

**Published:** 2019-08-22

**Authors:** Kristen N. Morrow, Craig M. Coopersmith, Mandy L. Ford

**Affiliations:** ^1^Immunology and Molecular Pathogenesis Program, Laney Graduate School, Emory University, Atlanta, GA, United States; ^2^Department of Surgery, Emory University School of Medicine, Atlanta, GA, United States; ^3^Emory Critical Care Center, Emory University School of Medicine, Atlanta, GA, United States; ^4^Emory Transplant Center, Emory University School of Medicine, Atlanta, GA, United States

**Keywords:** sepsis, IL-17, IL-33, critical illness, cytokine, immunological dysfunction, IL-27

## Abstract

Sepsis is a major cause of morbidity and mortality worldwide despite numerous attempts to identify effective therapeutics. While some sepsis deaths are attributable to tissue damage caused by inflammation, most mortality is the result of prolonged immunosuppression. *Ex vivo*, immunosuppression during sepsis is evidenced by a sharp decrease in the production of pro-inflammatory cytokines by T cells and other leukocytes and increased lymphocyte apoptosis. This allows suppressive cytokines to exert a greater inhibitory effect on lymphocytes upon antigen exposure. While some pre-clinical and clinical trials have demonstrated utility in targeting cytokines that promote lymphocyte survival, this has not led to the approval of any therapies for clinical use. As cytokines with a more global impact on the immune system are also altered by sepsis, they represent novel and potentially valuable therapeutic targets. Recent evidence links interleukin (IL)-17, IL-27, and IL-33 to alterations in the immune response during sepsis using patient serum and murine models of peritonitis and pneumonia. Elevated levels of IL-17 and IL-27 are found in the serum of pediatric and adult septic patients early after sepsis onset and have been proposed as diagnostic biomarkers. In contrast, IL-33 levels increase in patient serum during the immunosuppressive stage of sepsis and remain high for more than 5 months after recovery. All three cytokines contribute to immunological dysfunction during sepsis by disrupting the balance between type 1, 2, and 17 immune responses. This review will describe how IL-17, IL-27, and IL-33 exert these effects during sepsis and their potential as therapeutic targets.

## Introduction to Sepsis and the IL-17/IL-27/IL-33 Axis

Although it was first described centuries ago, sepsis remains a leading cause of morbidity and mortality. While the infectious agent and the organ system(s) impacted can vary between patients, sepsis is characterized by immune dysfunction linked to alterations in systemic cytokine levels and lymphocyte apoptosis (1). The immune response during sepsis was originally thought to proceed through two distinct phases through which an initially hyper-inflammatory immune response shifted toward profound immunosuppression caused by lymphocyte impairment (2). However, this only reflects the phenotype of circulating lymphocytes in some immunocompetent patients (3–6) and does not reflect the immune response in immunocompromised patients (7). In addition, evidence now exists that both pro- and anti- inflammatory cytokines are released shortly after sepsis onset (8, 9) and continue to be released in tandem throughout the course of the illness (10–12).

While there have been many positive animal studies demonstrating the beneficial effect of targeting cytokines during sepsis, this has not translated into improvements in clinical treatment; no clinical trials so far have led to an approved therapeutic. The reasons behind this are multifactorial and partially stem from a failure to consider the interaction between individual cytokines and the larger cytokine milieu. In addition, the cytokine milieu varies between septic patients, making it difficult to distinguish any benefit in large studies of heterogenous patients. Although the failure of cytokine-based therapy in septic patients has been disappointing, recent phase I clinical trials (such as IL-7 infusion) have demonstrated the potential benefit of immunomodulation (13). However, before additional cytokines can be considered as therapeutic targets for sepsis, further work needs to be done to define the alterations that occur across the cytokine milieu during sepsis and distinguish how individual cytokines interact and modulate the effects of one another.

Recent work on the cytokines IL-17, IL-27, and IL-33 suggest the presence of a novel cytokine axis during sepsis. IL-17 primarily acts to promote the inflammatory response in mucosal tissue. In humans, serum levels of IL-17 are predictive of the development of sepsis and mortality in poly-trauma patients (14) and mutations in the IL-17A gene are associated with increased susceptibility to infection caused by gram positive bacteria and mortality (15). The immunosuppressive cytokine IL-27 increases in the plasma of many septic patients (16–27) and has been shown to inhibit the differentiation of Th17 cells (16, 18, 19, 28–33). These results have been recapitulated in diverse models of sepsis in mice (34–38). The blockade of the p28 subunit of IL-27 (38) or depletion of IL-27 using a soluble and recombinant IL-27Rα (34) significantly reduces mortality in the cecal ligation and puncture (CLP) model of sepsis and is associated with reduced bacterial burden in the tissues and blood. IL-33 is a member of the IL-1 family of cytokines that modulates Th2 responses and decreases the differentiation of T cells into Th17 cells (39). IL-33 signals through the cytokine receptor ST2 and plays an anti-inflammatory role during sepsis, improving survival during the early stages of sepsis but ultimately leading to long lasting immunosuppression through the induction of regulatory T cells (Tregs) (40–42). In addition to its interactions with IL-17, IL-33 has also been reported to interact with IL-27, with both modulating the activity of ILC2 cells (43–46). As the importance of ILC2 cells during sepsis has recently been described (47–49), these interactions may become increasingly significant for the development of effective therapeutics.

In this review, we will further discuss the individual and combined roles of IL-17, IL-27, and IL-33 during sepsis and how this axis might be therapeutically targeted.

### The Role of IL-17 in Sepsis Pathophysiology

The IL-17 family of cytokines is composed of the structurally similar IL-17A-F. Apart from IL-17A (classically referred to as IL-17) and IL-17F, all the cytokines in this family are encoded separately, although they share conserved sequences. The earliest studies addressing the role of IL-17A during sepsis in animal models reported that they induced significant pathology and that eliminating IL-17A resulted in significantly improved survival (50, 51). However, subsequent studies using mice deficient in the IL-17 receptor found opposite results (52), and the literature now contains numerous studies demonstrating the mixed effects of IL-17A blockade in sepsis.

In 2012, Ogiku et al. reported that mice lacking IL-17A had significantly increased mortality following CLP that correlated with higher bacteremia at 12 h (53). Similarly, a more recent paper using the CLP model concluded that IL-17 has a partially protective role during sepsis: wild type mice had significantly increased survival and IgA production after CLP when compared to IL-17^−/−^ mice (54). Interestingly, this study found that non-canonical signaling through NF-κB was responsible for much of the IL-17A production, as mice lacking RANKL and NF-κB inducing kinase (NIK) signaling in their intestinal epithelium cells had significantly reduced IL-17A and mortality similar to IL-17^−/−^ mice (54).

Other studies have found that the impact of IL-17A on sepsis mortality depends on the microbe that initiated the infection. Using a bacterial pneumonia model, Ritchie et al. found that the role of IL-17A in sepsis is highly dependent on the encapsulation status of the infecting bacterium (55). IL-17A was beneficial during infections caused by minimally encapsulated bacteria, but significantly increased lung pathology and mortality if the infectious organism was heavily encapsulated (55). The authors concluded that this was due to the accumulation of neutrophils unable to phagocytose the bacteria (55). In conjunction with IL-23 signaling, IL-17A increases the recruitment of neutrophils and their accumulation in the lung following CLP, partially explaining the inflammation seen in the lung following polymicrobial sepsis originating in other tissues (56). IL-17 has also been linked to the development of acute kidney injury in septic patients and animal models (57). Given these findings, it is not surprising that multiple groups have reported that the neutralization of IL-17A or IL-17F improves survival (58, 59).

As the results of these sepsis studies conflict, it is important to note that IL-17A can induce the production of other IL-17 family cytokines, especially IL-17C (60). Although it is a distinct cytokine, it plays a similar role in neutrophil recruitment and the inflammatory process to IL-17A (60). In a mouse model of pneumonia induced by *Pseudomonas aeruginosa*, mice lacking IL-17C had 100% survival at 48 h, whereas wild type mice had only 25% survival at this time point (60). In contrast, another recent paper reported that IL-17C induction provides protection against LPS-induced endotoxemia (61). As IL-17C has been reported promote the production of IL-17A by Th17 lymphocytes in inflammatory conditions (such as autoimmune disease) (62), the authors concluded that these effects may be due more to the promotion of IL-17A than to IL-17C alone.

### The Role of IL-27 in Sepsis Pathophysiology

Originally thought to be pro-inflammatory, there is now consensus that IL-27 is a potent immunosuppressant. It is composed of an alpha subunit (IL-27p28, also known as IL-30) and EBI3 (shared with IL-35) (63). IL-27 binds to the IL-27 receptor alpha (IL-27Rα, also known as WSX-1) and gp130 and is primarily produced by dendritic cells (DCs), monocytes and macrophages (63). The lymphocyte populations that respond to the presence of IL-27 or one of its subunits are T cells, natural killer (NK) cells, natural killer T (NKT) cells, and DCs (64–69). This allows IL-27 to have wide ranging effects on cells of both the innate and adaptive immune response in addition to autocrine effects.

In septic patients and in murine models of sepsis, the plasma concentration of IL-27 significantly increases (34, 35, 70, 71), briefly causing it to be considered as a potential diagnostic biomarker in adults (22–24) and children (20, 26). However, these results have not been consistently replicated in humans, limiting its current therapeutic potential. In mice, the results are more consistent and indicate a clear role for IL-27 in the pathology of sepsis and critical illness. When the p28 subunit is neutralized or the IL-27Rα is blocked, mortality is significantly decreased in both CLP and endotoxemia (34, 37, 38).

In a study by Cao et al., mice lacking the IL-27Rα were resistant to a secondary bacterial infection caused by *Pseudomonas aeruginosa* following CLP in a manner dependent on alveolar macrophages and neutrophils (37). Specifically, the neutrophils and alveolar macrophages in these mice had a significantly improved ability to kill *P. aeruginosa* upon phagocytosis (37). Similarly, Bosmann et al. observed that the oxidative burst of macrophages was improved upon the elimination of IL-27 signaling, and determined that IL-10 limits the production of IL-27p28 *in vivo* following CLP (38). In addition, this study found that the cells primarily responsible for the production of IL-27p28 in the CLP model of sepsis are splenic macrophages (38). However, a more recent study has found conflicting evidence that indicates a protective role for p28 during sepsis (72). In this study, the administration of the p28 subunit or its overproduction through genetic therapy led to a reduction in mortality during sepsis directly linked to the reduction in NKT cell production of inflammatory cytokines (72).

In addition to its modulation of innate cells, IL-27 has a significant impact on T cells. IL-27 can promote the differentiation of Th1 cells and it is also a potent inducer of type 1 Treg (Tr1) cells (73). While Tr1 cells produce IFN-γ, they also produce large quantities of IL-10 and have potent suppressive functions (74). In addition to the induction of this cell population, IL-27 signaling leads to an increase in co-inhibitory molecule expression on T cells following chronic antigen exposure and during cancer (75). As T cell dysfunction and exhaustion is associated with the development of immunosuppression during sepsis and ultimately worsened survival (76–79), IL-27 could be an effective therapeutic target. However, mice can produce IL-27p28 in the absence of EBI3, so it is unclear if the reported effects of IL-27 during sepsis are actually due to the full heterodimeric cytokine or merely to its alpha subunit. A group has recently reported the development of transgenic B57L/6J mice in which the IL-27p28 subunit cannot be produced independently of EBI3 (80). This animal model will be necessary to truly distinguish the effects of IL-27p28 from those of IL-27.

### The Role of IL-33 in Sepsis Pathophysiology

ST2 was an orphan receptor until 2005, when Schmitz et al. reported their discovery of IL-33 (81). A member of the IL-1 family, IL-33 is constitutively expressed by endothelial and epithelial cells in barrier tissues and is also found at high levels under inflammatory conditions in other tissues (82, 83). When T cells, mast cells, eosinophils, and ILC2s receive IL-33 signaling, the immune response shifts toward a type 2 response (81, 84).

The first paper to describe the role of IL-33 (rather than its receptor ST2) in sepsis was published by Alves-Filho et al. (40). The authors found that survival significantly increased when IL-33 was administered to mice following CLP (40). Another 2010 study found that IL-33 is protective against LPS induced endotoxemia (85). The ability of IL-33 to improve survival during sepsis is linked to the rescue of neutrophil migration to the site of infection (40), to improvements in bacterial clearance, and to a reduction in lymphocyte apoptosis (41). IL-33 also suppresses the inflammatory response by a variety of innate lymphocytes (86) and modulates the activity of ILC2 cells (47–49). In addition to direct effects on other lymphocytes, IL-33 impacts the activity of other cytokines, including IL-17 (41, 87). While IL-33 can bind to a soluble form of ST2 (sST2), the effects of IL-33 during sepsis appear to be dependent on signaling through membrane bound ST2; in one study, patients who had did not survive sepsis had higher levels of sST2 than patients that went on to survive their infections (40).

Despite being linked to improvements in survival early after sepsis onset, IL-33 signaling may not always be beneficial. IL-33 is implicated in the induction and maintenance of immunosuppression during sepsis through the induction of Tregs (42). Nascimento et al. found that this occurs through the production of IL-4 and IL-13 by ILC2s that receive IL-33 signaling (42). The IL-4 and IL-13 then drives the proliferation of IL-10 producing macrophages and ultimately an expansion in Treg numbers (42). When they examined the blood of a small number of patients who had been diagnosed with sepsis 5–10 months prior, they found that sepsis survivors had significantly higher concentrations of both IL-10 and IL-33 and higher circulating Treg numbers compared to previously healthy patients (42). While these findings need to be replicated, they suggest that the impact of IL-33 signaling may depend on the stage of disease.

## Interactions Between IL-17, IL-27, and IL-33

**Figure 1:**
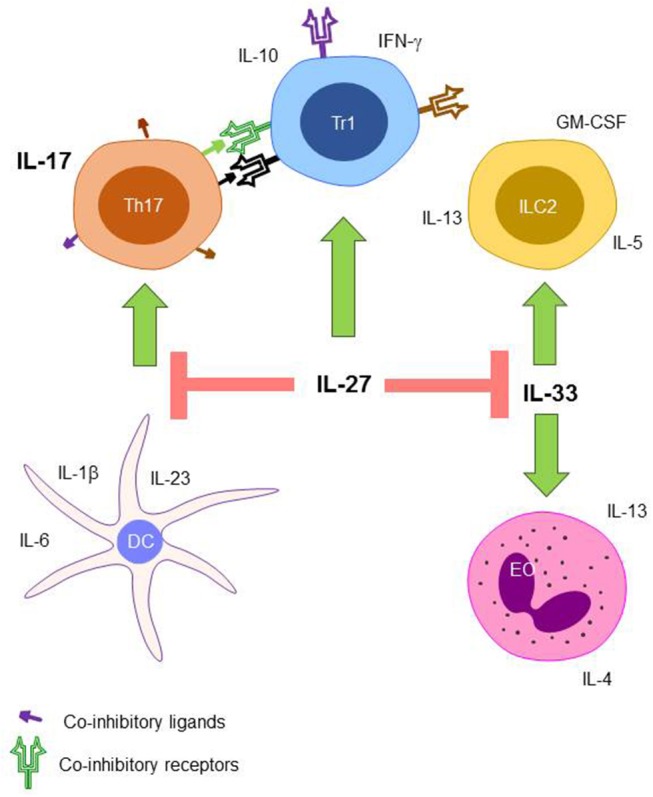
The proposed role of the IL-17, IL-27, and IL-33 axis during sepsis. Th17 cells that receive co-inhibitory signaling from IL-27 induced Tr1 cells have inhibited production of IL-17. Differentiation of naïve T cells into Th17 cells is also inhibited by IL-27 through the modulation of DC cytokine production. ILC2 cells and EOs expansion are also inhibited through the action of IL-27 signaling on IL-33. Th17, T helper type 17 cell; Tr1, T regulatory type 1 cell; ILC2, innate lymphocyte type 2 cell; DC, dendritic cell; EO, eosinophil.

The ability for lymphocytes to recognize and respond to slight changes in their environment makes the immune system very adaptable and ensures that the balance between inflammatory and immunosuppressive responses is fine-tuned. While the ability of lymphocytes to respond so readily to their surroundings is beneficial from an evolutionary point of view, it makes it significantly harder to elucidate the role of individual cytokines. The individual and combined actions of the cytokines in the IL-17, IL-27, and IL-33 axis are summarized in Figure 1.

### IL-17 and IL-27

IL-17 plays a harmful role in many autoimmune diseases, particularly experimental autoimmune encephalomyelitis (EAE) and rheumatoid arthritis (RA). By limiting the differentiation of naïve CD4^+^ T cells into Th17 cells, IL-27 is able to attenuate these diseases (16, 19, 28, 31, 32, 88). Similarly, IL-27 signaling prevents the development of neurological damage during chronic *Toxoplasma gondii* infection (18) and reduces tissue damage during RSV infection (89). Further research has shown that STAT1 signaling (which IL-27 induces) inhibits the expression of the transcription factor RORγt, necessary for Th17 differentiation, while promoting the induction of the protein suppressor of cytokine signaling 1 (SOCS1) (16, 19, 30, 90). This leads to the suppression of IL-22 production by Th17 cells, impairing antimicrobial defenses in the epithelium (30, 33). In addition to its direct effects on T cells, IL-27 can also inhibit Th17 differentiation by inhibiting the production of the Th17-polarizing cytokines IL-1β, IL-6, and IL-23 by DCs (29). In contrast, T cells that have already committed to the Th17 lineage are not directly inhibited by IL-27 signaling (28, 91). Instead, inhibition occurs indirectly through the induction of Tr1 (29) and the expression of co-inhibitory receptors and their ligands (32, 88).

### IL-17 and IL-33

Similar to IL-27, IL-33 has been reported to attenuate EAE through the suppression of Th17 responses (92). While IL-33 attenuates sepsis mortality, it is less clear if this is due to any effect on the Th17 response. One group reported that the administration of IL-33 actually enhanced the production of IL-17 while decreasing the levels of IL-6, IL-10, and IFNγ following CLP (41). Similarly, another group found that the deletion of the IL-33 receptor ST2 led to a reduction in the frequency and number of IL-17 producing NK cells after CLP (86). However, a recent study of human patients with *Staphylococcus aureus* bacteremia revealed that a higher ratio of Th17 to Th1 cytokines early after sepsis onset was associated with increased mortality (49). As there was a trend toward increased Th2 cells in surviving patients, the authors did a follow up study using a mouse model of *S. aureus* bacteremia (49). IL-33 provided a survival benefit in this model that was dependent on functional ILC2s and EOs, suggesting that IL-33 is protective in part because it re-balances type 1, 2, and 17 responses during sepsis (49).

### IL-27 and IL-33

While IL-27 signaling promotes type 1 immune responses and directly limits type 17 immunity, it also serves as a negative regulator of type 2 responses by interfering with IL-33 signaling. The first paper to describe this phenomenon utilized *in vitro* experiments which showed that IL-27 reduced type 2 cytokine production in bone marrow cells exposed to IL-33, including IL-5, IL-13, and GM-CSF (43). For IL-5, this effect was dependent on STAT1 signaling, as STAT1 knockout bone marrow cells were not impacted by the presence of IL-27 (43). Moro et al. confirmed these findings *in vivo* using STAT1 knockout mice, and revealed that while IL-27 reduces type 2 cytokine production by ILC2 cells, it does not affect cytokine production in Th2 cells (44). Another recent study reported that the administration of IL-27 limits IL-33 induced ILC2 accumulation and activation in the lungs, liver, spleen, and mesenteric lymph node *in vivo* (45). The administration of IL-27 also led to the overrepresentation of IL-27Rα^−/−^ cells in chimeric mice (45). While not specifically addressing IL-27, another murine study found that STAT1 signaling induced by infection with respiratory syncytial virus is sufficient to reduce the production of IL-33 (46). These studies collectively show that a major function of IL-27 is to negatively regulate the type 2 immune response, specifically ILC2 cells, in a manner that is dependent on STAT1 signaling.

## Exploiting the IL-17, IL-27, and IL-33 Axis During Sepsis

While many reviews have discussed the therapeutic potential for targeting IL-17, IL-27, and IL-33 during sepsis (93–95), none have considered the effect that treatment targeting only one of these cytokines may have on the others. In addition, the compartmentalization of the immune response during sepsis means that cytokine therapies that restore the function of circulating lymphocytes could cause excessive stimulation and ultimately programmed cell death in the more normally responsive tissue lymphocytes. To aid the specificity of these therapies, binding should be targeted to cells expressing the circulatory chemokine receptor molecule CCR7 or the integrin CD62L (required for lymphocyte extravasation into the lymphatic system).

As IL-17 can have either beneficial or detrimental roles during sepsis depending on the murine model used, it is currently unclear what course of action would be most beneficial for human patients. However, anything that significantly increases IL-17 levels for a long period of time raises the risk of auto-immune disease formation and increased tissue damage. It seems more tenable to target IL-27 and IL-33, with an eye to keeping a balance between these cytokines and IL-17.

Neutralizing IL-27 seems likely to provide a survival benefit in septic patients if administered early after disease onset. IL-27 signaling shifts the balance too far toward a type 1 regulatory response, but its neutralization would balance type 1 and type 2 responses through the increase in activity of the type 2 promoting cytokine IL-33. IL-33 signaling has been shown to improve sepsis survival in the short term in murine models, although one report suggests that IL-33 is linked to the development of immunosuppression during sepsis (42). In this study, mice lacking the IL-33 receptor had attenuated immunosuppression associated with a reduction in type 2 cytokines, ILC2 cells, and Tregs (42). It is currently unknown how much IL-33 signaling changes during sepsis in the absence of IL-27 signaling and therefore might lower the efficacy of IL-27 blockade in improving long term survival in sepsis patients who receive no further interventions.

Ultimately, targeting any of these cytokines in an indiscriminate fashion is unlikely to be clinically beneficial. However, understanding the complex interplay between IL-17, IL-27, and IL-33—including the timing in which cytokine augmentation or blockade may potentially be beneficial—suggests this axis may potentially be manipulatable for therapeutic gain as part of a precision medicine approach toward sepsis treatment.

## Author Contributions

KM wrote the initial draft of the manuscript. The final manuscript includes equal contribution from MF and CC.

### Conflict of Interest Statement

The authors declare that the research was conducted in the absence of any commercial or financial relationships that could be construed as a potential conflict of interest.
